# Supplementary figures and images for: A Simple Methodology to Estimate the Diffusion Coefficient in Pervaporation-Based Purification Experiments
Source: Polymers (Basel). 2019 Feb 15;11(2):343. doi: 10.3390/polym11020343 (PMC6419206; doi:10.3390/polym11020343)

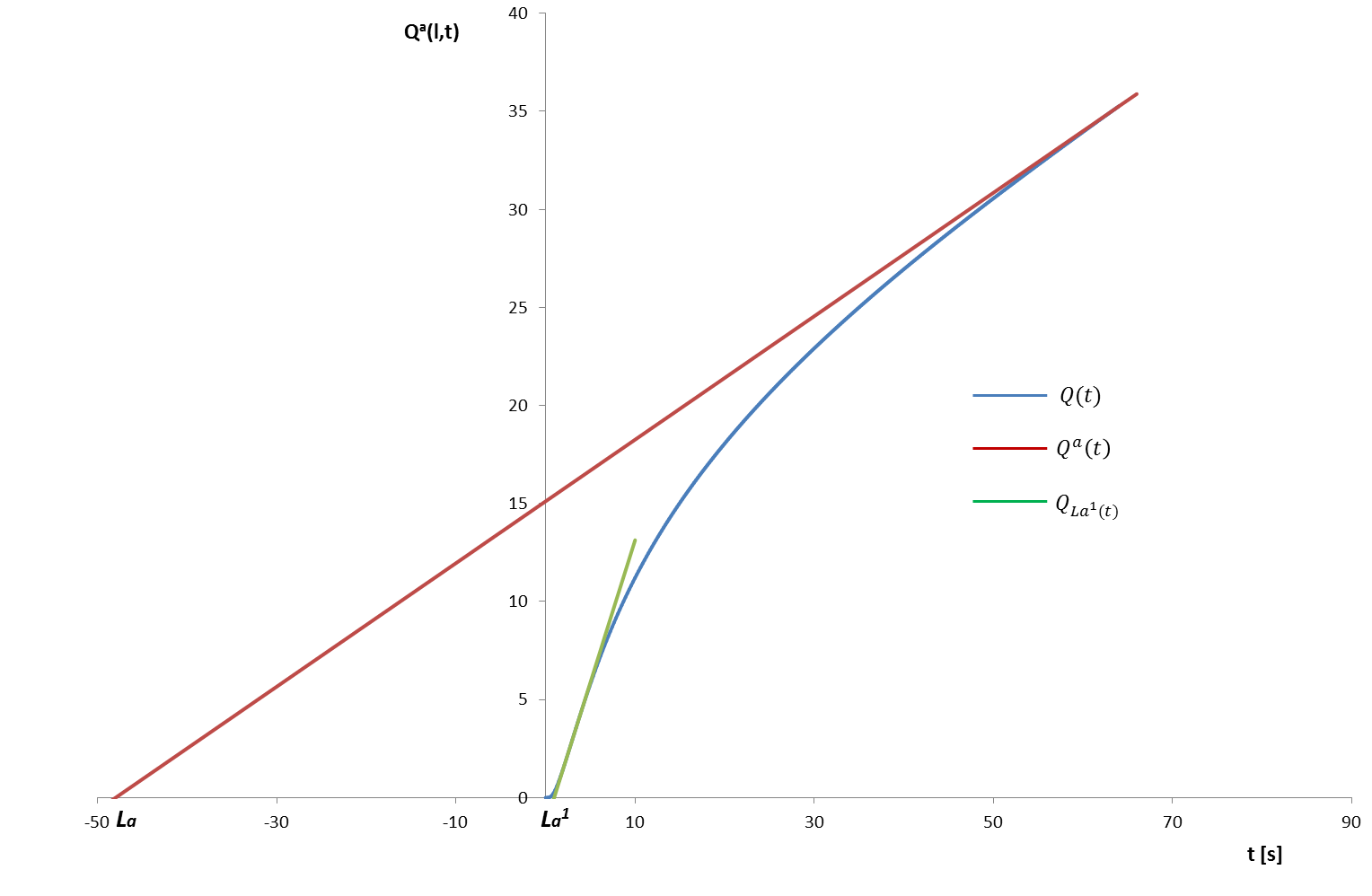

Supplement: Supplementary file 1 [file polymers-11-00343-s001.zip › polymers-440569-supplementary.tif]
